# Crocin protects the renal tubular epithelial cells against high glucose-induced injury and oxidative stress via regulation of the SIRT1/Nrf2 pathway

**DOI:** 10.22038/IJBMS.2022.51597.11708

**Published:** 2022-02

**Authors:** Jichen Zhang, Xuemei Zhao, Hongling Zhu, Jingnan Wang, Junhua Ma, Mingjun Gu

**Affiliations:** 1 Department of Endocrinology, Shanghai Pudong New District Gongli Hospital, Second Military Medical University, Shanghai 200135, P. R China; 2 Postgraduate Education College, Ningxia Medical University, Yinchuan, Ningxia 750004, P.R. China

**Keywords:** Diabetic nephropathy, High glucose, NF-E2-related factor 2, Oxidative stress, Renal tubular epithelial cell Sirtuin 1

## Abstract

**Objective(s):**

Renal tubular damage is critical pathological feathers of diabetic nephropathy (DN). This study aimed to explore the protective activity and related mechanisms of crocin in renal epithelial cell injury induced by high glucose.

**Materials and Methods:**

Renal tubular epithelial HK-2 cells were cultured with D-glucose to establish an in vitro DN model. Cell viability was evaluated by CCK-8 assay. Apoptosis was detected by Annexin V-FITC kit. Oxidative stress was evaluated by colorimetry. RT-qPCR was carried out to determine the mRNA expressions of NF-E2-related factor 2 (Nrf2) and its pathway genes. Western blot was applied to determine the protein expressions of Nrf2 and related proteins.

**Results:**

High glucose (5.5, 30, and 50 mM D-glucose) decreased cell viability at 72 hr, which was attenuated by crocin (25 and 50 μM). Crocin also attenuated the high glucose (30 mM D-glucose) induced apoptosis of HK-2 cells, decreased MDA content, and increased SOD activity in culture media. Crocin increased mRNA levels of Nrf2, HO-1, and NQO1. Moreover, crocin increased protein expressions of Nrf2, Sirtuin 1 (SIRT1), and p-Akt (Ser473). Inhibition of Nrf2 using siRNA, and inhibitors of SIRT1 (nicotinamide, NAM, 20 μM) and PI3K/Akt (LY294002, 50 μM) all attenuated the protective effect of crocin. Nrf2 siRNA and NAM also partially attenuated the inhibitory effect on oxidative stress and increase in the Nrf2 protein by crocin treatment.

**Conclusion:**

Crocin protects renal epithelial cells against injury induced by high glucose, and the mechanism is associated with partial activation of the SIRT1-Nrf2 pathway.

## Introduction

Diabetic nephropathy (DN) is a progressive microvascular complication of diabetes mellitus and is one of the leading causes of DN patients (1). The pathology of DN is characterized by basement membrane thickening, extracellular matrix accumulation, and subsequent albuminuria (2). DN is driven by numerous factors, among which hyperglycemia alters cellular signaling and metabolism, contributes to glomerular hyperfiltration (3), increases advanced glycation end products (AGEs) (4), oxidative stress (5), and inflammation (6).

Oxidative stress is considered an important pathological process that links hyperglycemia to vascular complications through changing renal metabolism and hemodynamics, thereby leading to adverse effects on kidney tissues (7). Excessive oxidative stress could offset the endogenous antioxidant defense system, and then oxidize various biomolecules like DNA, proteins, carbohydrates, and lipids (8). Hyperglycemia aggravates oxidative stress through various mechanisms (9), which involves the progression of diabetic complications (10).

Crocin is one major pharmacologically active constituent of saffron (*Crocus sativus L*.). Crocin has demonstrated various pharmacological effects, including anti-inflammatory, anticonvulsant, and anti-tumor activities (11), and it is reported to inhibit cisplatin-induced oxidative stress in rats (12). Current studies have shown that crocin exhibits significant antioxidant activity and scavenges ROS (13), and this may be related to its endogenous antioxidant enzymatic activities (14).

In this study, we established an *in vitro* injury model using HK-2 human renal tubular epithelial cells, explored the protective effect of crocin on high glucose-induced injury, and evaluated cell viability, apoptosis, oxidative stress, and related molecular mechanisms.

## Materials and Methods

***Cell culture***

The HK-2 cells were purchased from the Chinese academy of sciences. HK-2 cells were cultured in a low glucose DMEM medium (5.5 mM D-glucose) with 10% FBS and penicillin/streptomycin (HyClone). Cells were cultured in a 5% CO_2_ incubator at 37 °C.

***Experiment grouping***

The HK-2 cells were seeded in 96 well culture plates (2×10^4^ cells per well, in 100 μl DMEM medium). When cells grew to 80% confluence, they were divided into the following groups: (1) Control group: cells were incubated with only 5.5 mM D-glucose (normal glucose); (2) High-glucose group: cells were incubated with D-glucose at 15, 30, and 50 mM for up to 72 hr; (3) Crocin group: cells were incubated with 30 mM D-glucose and crocin at 5, 10, 25, 50, and 100 μM for 48 and 72 hr.

***Cell viability assay***

After various treatments, HK-2 cells were added with CCK-8 solution (10 μl; Beyotime, Shanghai, China), and were incubated at 37 ^°^C for 2 hr. The absorbance at 450 nm was measured using a microplate reader.

***Apoptosis assay***

The HK-2 cells were seeded in 24-well plates (3×10^5^ cells/well) and cultured in a medium containing 30 mM D-glucose, followed by treatment with crocin 25 and 50 μM for 72 hr. Cells were collected and washed with cold PBS and were incubated with 5 µl Annexin V-FITC and 5 µl PI in the dark for 15 min. Apoptosis was assessed by a flow cytometer (Becton Dickinson, San Francisco, CA, USA).

***Oxidative stress test***

 Following the treatment, HK-2 cells were lysed using the RIPA lysis buffer. The supernatant was collected for the experiments. The oxidative and antioxidant proteins were measured by spectrophotometry. (Nanjing Jiancheng Bioengineering Research Institute, Nanjing, China). The determination of malondialdehyde (MDA) (cat no. S0131S) and superoxide dismutase (SOD) activity (cat no. S0109) were investigated by commercial assay kits (Beyotime, Jiangsu, China). Eventually, MDA activity was measured with a microplate reader at 532 nm absorbance and expressed as μmol/l. SOD activity was measured at 520 nm absorbance and expressed as U/L.

***siRNA transfection***

 The Nrf2 small interference RNA (siRNA) was designed and synthesized by Shanghai GenePharma Co., Ltd, using the following primer: 5’-CAC ACT GGA TCA GAC AGG AGG ATA T-3’ for transfection. Following the manufacturer’s instructions, the siRNA was transfected into the HK-2 cells with Lipofectamine® 2000 transfection kit (Invitrogen, USA). Briefly, the 5x10^5^ HK-2 cells were seeded into the 6-well plates in a complete medium and transfected by 100 pmol siRNA and 5 μl of Lipofectamine® 2000 per well. After 36 hr of incubations, cells were further treated with high glucose and crocin and used for the experiments.

***RT-qPCR***

Total RNA was extracted by Trizol agent and was used to generate cDNA by the Superscript III enzyme (Life Technologies). RT-qPCR was carried out using SYBR® Premix Ex Taq™ II (cat. no. RR820L; Takara Bio, Inc.). The PCR conditions were as follows: denaturation at 94 ^°^C for 5 min; followed by 40 cycles at 94 ^°^C for 5 sec and 60 ^°^C for 60 sec. PCR primers for Nrf2, HO-1, NQO1, and GAPDH were listed in Table 1. Reaction systems contained 2 μl template cDNA, 10 μl SYBR-Green PCR master mix, 1 μl forward and reverse primers, and 7.2 μl deionized water. PCR results were quantified by using the 2−ΔΔCt method.

***Western blotting***

Total protein was extracted from HK-2 cells using RIPA buffer. Protein samples (50 μg) were subjected to 12% SDS-PAGE and then transferred to a PVDF membrane (Millipore, Bedford, USA) for detection with appropriate antibodies. Membranes were blocked with 5% nonfat dry milk in TBST buffer and treated with primary antibodies against human Nrf2 (#12721), SIRT1 (#9475), and p-Akt1 (Ser473) (#4060) (all 1:1000 dilutions; Cell Signaling Technology, USA) at 4 ^°^C overnight, washed and then incubated with HRP-linked secondary antibody (1:1000 dilution) for 1 hr at room temperature. A chemiluminescent detection system (ECL, UK) was utilized for visualization. The gray value of the target band was normalized to that of β-actin.

***Statistical analysis***

Data from at least three independent experiments were presented as means ± standard deviation (SD), and analyzed by SPSS 20.0 statistical software (SPSS Inc., Chicago, IL, USA). Unpaired Student’s t-test or ANOVA was used to analyze the differences between two or more groups. *P*<0.05 was considered as statistical significance.

## Results

***High glucose decreased cell viability of high glucose-induced HK-2 cells***

**Figure 1:**
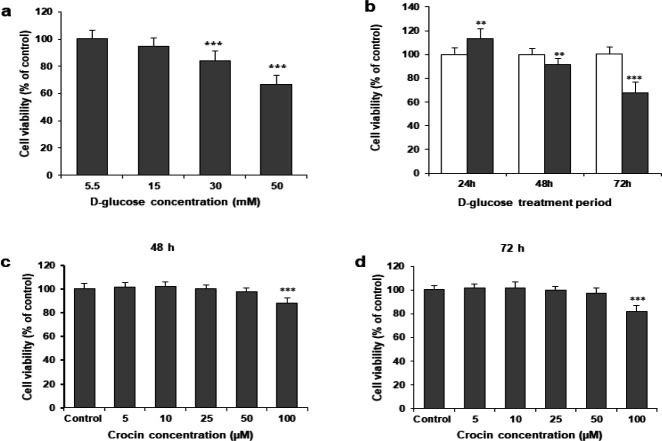
High glucose decreases cell proliferation of renal tubular epithelial cells. (a) Cell viability of HK-2 cells that were incubated with 5.5, 15, 30, or 50 mM D-glucose for 72 hr. *P<0.05, **P<0.01, and ***P<0.001 vs 5.5 mM group. (b) Cell viability of HK-2 cells with 5.5 or 50 mM glucose for 24, 48, or 72 hr. ***P<0.001 vs the 5.5 mM group at each time point. HK-2 cells were treated with crocin (5, 10, 25, 50, and 100 μM) for 48 (c) or 72 hr (d). Low concentrations of crocin had little cytotoxic effects, while high concentrations of crocin significantly reduced the viability of HK-2 cells. *P<0.01 and **P<0.001 vs control group

HK-2 cells were incubated with D-glucose (5.5, 15, 30, or 50 mM) for 72 hr. D-glucose treatment at 15, 30, and 50 mM significantly reduced cell viability (Figure 1a). Then HK-2 cells were incubated with 5.5 and 30 mM D-glucose for different time points. Compared to cells with normal glucose, 30 mM D-glucose markedly reduced viability at 48 and 72 hr (Figure 1b). We then examined the toxicity of crocin at various concentrations. Crocin had little effect on cell viability between 10, 25, and 50 μM at 48 and 72 hr. However, crocin at 100 μM dramatically reduced cell viability at 48 and 72 hr (Figures 1c, 1d). Thus, we chose crocin at 25 and 50 μM for further experimental analysis.

***Effect of crocin on cell viability and apoptosis in high glucose-induced HK-2 cells***

**Figure 2:**
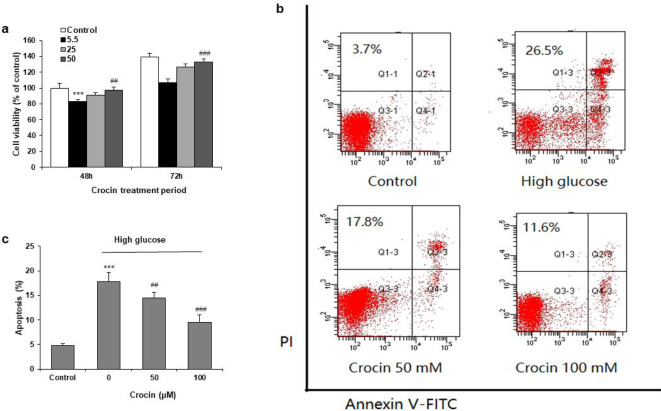
Inhibitory effects of crocin on high glucose-induced cell viability reduction and apoptosis. (a) HK-2 cells were treated with high glucose with or without crocin (25 and 50 μM) for 48 or 72 hr. (b) Apoptosis was evaluated by Annexin-V FITC and PI double staining using flow cytometry after crocin treatment for 72 hr. The apoptotic rate was calculated by the cell number of the lower right quadrant normalized to that of the lower-left quadrant plus the lower right quadrant. (c) High glucose increased apoptotic rate in high glucose-induced HK-2 cells was significantly attenuated by crocin at 25 and 50 μM. **P<0.01 and ***P<0.001 vs Control group; ^##^*P<*0.01 and ^###^*P<*0.001 vs high glucose group

HK-2 cells were incubated with 30 mM D-glucose, followed by crocin treatment (25 and 50 μM) for 48 and 72 hr. Crocin significantly attenuated the D-glucose-induced decrease in viability at 48 and 72 hr (*P*<0.05) (Figure 2a). In order to explore whether apoptosis mediates reduced cell survival by crocin, Annexin V/PI staining was performed. High glucose (30 mM D-glucose) markedly induced apoptosis at 72 hr, and crocin at 25 and 50 μM both significantly attenuated the increase in apoptotic rate induced by high glucose (Figures 2b, 2c).

***Crocin activated Nrf2 pathway and inhibited oxidative stress in high glucose-induced HK-2 cells***

**Figure 3:**
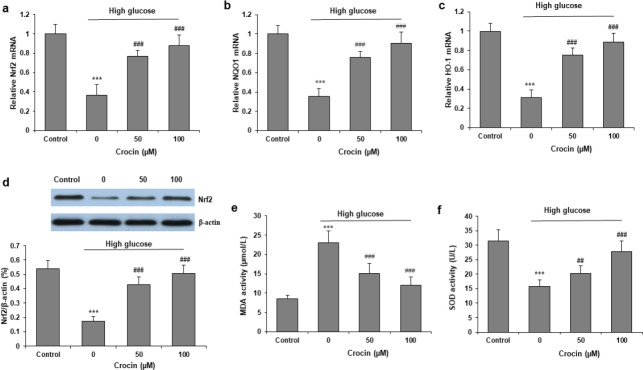
Crocin activates the Nrf2 pathway and inhibits oxidative stress in high glucose-induced cells. HK-2 cells were treated with high glucose with or without crocin (25 and 50 μM) for 72 hr. RT-qPCR was carried out to determine mRNA levels of Nrf2 (a), HO-1 (b), and NQO1 (c). Western blot was carried out to determine the protein level of Nrf2 (d). MDA content (e) and SOD activity (f) were measured in culture media to evaluate oxidative stress. *P<0.05 and ***P<0.001 vs Control group; ^##^*P<*0.001 and ^###^*P<*0.001 vs high glucose group

Hk-2 cells were incubated with 30 mM D-glucose and crocin (25 and 50 μM) for 72 hr co-culture. High glucose markedly reduced the mRNA and protein levels of Nrf2. Co-treatment with crocin increased mRNA expressions of Nrf2 (Figure 3a), HO-1 (Figure 3b), and NQO1 (Figure 3c). Then Western blotting was performed and protein expression of Nrf2 was also increased by crocin (Figure 3d). We then investigated the effect of crocin on oxidative stress. Crocin markedly reversed 30 mM D-glucose increase in MDA content and decrease SOD activity in HK-2 cells (Figures 3e, 3f).

***Crocin protected HK-2 cells against high glucose-induced injury via the SIRT1-Nrf2 pathway***

**Figure 4:**
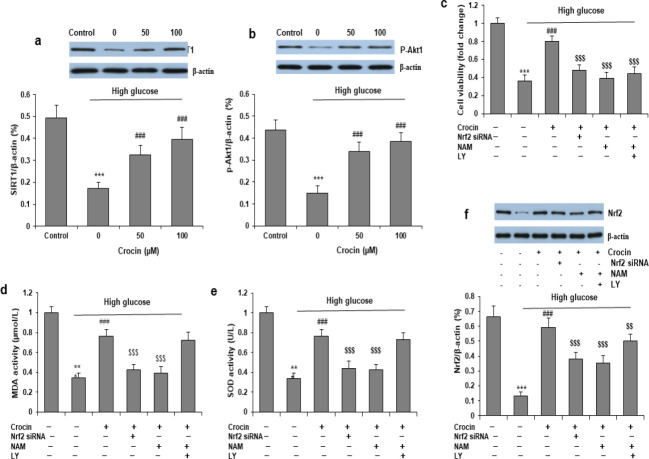
SIRT1 and Akt mediate the protective effects of crocin on high glucose-induced injury. Western blot was carried out to measure the protein levels of SIRT1 (a) and p-Akt1 (b). HK-2 cells were pre-incubated with Nrf2 siRNA, SIRT1 specific inhibitor nicotinamide (NAM, 20 μM), or PI3K inhibitor LY294002 (LY, 50 μM), then pretreated with high glucose, and then with or without crocin (25 and 50 μM) for 48 and 72 hr. Cell viability (c), MDA activity (d) and SOD activity (e), and Nrf2 protein expression (f) were determined at 72 hr. ***P<0.001 vs Control group; ^###^*P<*0.001 vs high glucose group; ^$$$^*P<*0.001 vs crocin group

Western blot was carried out to measure the protein expressions of SIRT1 and p-Akt1 (Ser473). 30 mM D-glucose significantly decreased SIRT1 and p-Akt1 (Ser473) expression in HK-2 cells, and these changes were partially attenuated by cotreatment with crocin (25 and 50 μM) (Figures 4a, 4b). To further investigate the role of SIRT1 and Akt1 in the protective effect of crocin against high glucose-induced injury, we knocked down Nrf2 expression by siRNA, and co-incubated HK-2 cells with SIRT1 specific inhibitor, nicotinamide (NAM, 20 μM), or with LY294002 (a specific inhibitor of PI3K/Akt1, 50 μM) before crocin treatment. Inhibition of Nrf2, SIRT1, or Akt1 significantly attenuated the protective effects of crocin (Figure 4c). Moreover, Nrf2 siRNA and SIRT1 partially attenuated the inhibition of crocin on oxidative stress, while LY294002 showed no significant effect on oxidative stress (Figures 4d, 4e). Furthermore, we explored the regulation between SIRT1, p-Akt1, and Nrf2. Nrf2 siRNA and NAM attenuated the increase in Nrf2 protein by crocin, while LY294002 partially attenuated the increase in Nrf2 protein, with a reduction rate of about 30% (Figure 4f). These data indicate that SIRT1 and Akt1 play critical roles in the protection of crocin in high glucose-induced cells injury, and SIRT1 is also an upstream regulator of Nrf2 and oxidative stress. 

**Table 1:** Primer sequences

Genes	Forward primer (5′-3′)	Reverse primer (5′-3′)
Nrf2	ACA CGG TCC ACA GCT CAT C	TGC CTC CAA GTA TGT CAA TA
HO-1	TTG CCA GTG CCA CCA AGT TC	TCA GCA GCT CCT GCA ACT CC
NQO1	GGA TTG GAC CGA GCT GGA A	GAA ACA CCC AGC CGT CAG CTA
GAPDH	GCA CCG TCA AGG CTG AGA AC	TGG TGA AGA CGC CAG TGG A

## Discussion

In this study, we found that crocin attenuated the high glucose-induced reduction in cell viability. Crocin also increased mRNA levels of Nrf2, HO-1, and NQO1, and suppressed oxidative stress in high glucose-induced HK-2 cells. Crocin increased protein expressions of SIRT1 and p-Akt (Ser473), and inhibition of SIRT1 (nicotinamide, NAM, 20 μM) and PI3K/Akt (LY294002, 50 μM) all attenuated the protective effect of crocin on high glucose-induced HK-2 cells. Therefore, the protective effect of crocin might be related to the modulation of Nrf2, SIRT1, and Akt molecules.

Crocin showed suppressive effects on high glucose-induced toxicity and oxidative injury. These results support the concept that high glucose is a potent inducer of oxidative stress, and thereby initiates and aggravates DN [9]. Our results are consistent with previous reports that crocin relieved DN and improved renal function in diabetic rats through inhibition of oxidative stress and inflammation (15, 16). Moreover, crocin also protects podocytes from high glucose-induced oxidative injury (17). Our study provides the same protective effect on renal tubular cells. Recently, changes in the renal tubules, are increasingly implicated in the development and progression of diabetic kidney disease (18). The renal tubules play the same important roles as glomeruli and podocytes in the pathogenesis of DN (19), and tubular injury has been postulated as a critical contributor to the early DN (20). Thus, our study provides another target tissue to elucidate the roles and mechanisms of crocin in DN.

This study showed that crocin activated Nrf2 and downstream antioxidant response in combating high glucose-induced injury. Nrf2 is an endogenous antioxidant defense system that maintains cellular homeostasis under stress conditions. Nrf2 could slow down the progression of DN (21). High glucose treatment inhibited Nrf2 expression in renal tubular epithelial cells, and overexpression of Nrf2 reduced renal cell damage in diabetes (22). Our study adds crocin as a new Nrf2 activator in high glucose-induced renal epithelial cells, which is supported by other reports that crocin modulates Nrf2 in hepatic ischemia-reperfusion injury (23) and cigarette smoke-induced lung injury (24).

Our study showed that crocin increased SIRT1 protein, which was essential for the protective effect of crocin in high glucose-induced injury. Sirtuin 1 (SIRT1) modulates NAD(+) coenzymes and maintains cellular energetic metabolism and oxidative state. SIRT1 deficits are associated with diabetes mellitus and kidney diseases. SIRT1 promoted the survival of kidney cells and protected against apoptosis in renal tubules (25). In renal tubular injury induced by hyperglycemia, SIRT1 activation could attenuate renal tubular injury through inhibiting apoptosis (26), which is consistent with our results that SIRT1 inhibition abolished the protective effect of crocin. Our study also showed that SIRT1 might lie upstream of Nrf2, as SIRT1 inhibition abolished the increase of Nrf2 protein by crocin. High glucose could induce injury of renal epithelial cells via activation of the SIRT1/NF-κB/Nrf2 pathway (27). The SIRT1/Nrf2 pathway was also activated by crocin and mediated alleviation of myocardial ischemia/reperfusion-induced injury (28). Our study firstly reported SIRT1/Nrf2 activation by crocin in high glucose-induced renal epithelial cells, and provided experimental data for further investigation on mechanisms of crocin in DN.

In the present study, crocin enhanced p-Akt1 protein, which was essential for protective effect on high glucose-induced injury. Akt1 is a signal protein that modulates many cellular processes such as glucose uptake, cell survival, and angiogenesis in the kidney, and its activation showed protective effects in DN (29), and also inhibited high glucose-induced apoptosis of renal epithelial cells (30). This can explain how Akt1 inhibition could abolish high glucose-induced cellular injury by high glucose. Our results are in accordance with other reports that crocin activated Akt1 in microglial cells of diabetic retinopathy (31) and myocardial ischemia/reperfusion injury (32). In these reports, Akt1 activation was associated with induction of autophagy, which protected podocyte function and showed a preventive effect on DN progression (33). However, it remains unclear about the role of autophagy in the protective effect of crocin on high glucose-induced renal tubular injury. Moreover, in our study Akt1 inhibition could not attenuate oxidative stress and only partially attenuated the increase in Nrf2 protein. This indicates that Akt1 activation by crocin may further activate pathways other than Nrf2, especially in cellular survival.

## Conclusion

Crocin attenuates high glucose-induced oxidative injury in renal tubular epithelial cells, which is related to the SIRT1-Nrf2 pathway. Crocin may be a promising therapeutic agent of DN, especially through targeting renal tubules against oxidative stress initiated by high glucose.

## Authors’ Contributions

JZ performed experiments and wrote the manuscript; XZ, HZ, and JW performed experiments and collected data; JM analyzed the data and revised the manuscript; MG designed and supervised the study. All authors have read and approved the manuscript.

## Funding

This work was supported by a project of discipline construction of health and family planning commission in Pudong New District: health committee in Pudong New District 2021 Health Science and Technology Project (PW2021A-33); and a pilot project of Traditional Chinese Medicine (TCM) care service in public hospitals for comprehensive reform of development of traditional Chinese medicine in Pudong New District (Nov 2015).

## Conflicts of Interest

The authors declared no conflicts of interest with other people or organizations.
